# A Passive Heat Maintenance Strategy Implemented during a Simulated Half-Time Improves Lower Body Power Output and Repeated Sprint Ability in Professional Rugby Union Players

**DOI:** 10.1371/journal.pone.0119374

**Published:** 2015-03-18

**Authors:** Mark Russell, Daniel J. West, Marc A. Briggs, Richard M. Bracken, Christian J. Cook, Thibault Giroud, Nicholas Gill, Liam P. Kilduff

**Affiliations:** 1 Department of Sport, Exercise and Rehabilitation, Health and Life Sciences, Northumbria University, Newcastle upon Tyne, United Kingdom; 2 Applied Sports Technology Exercise and Medicine Research Centre (A-STEM), Health and Sport Portfolio, Swansea University, Swansea, United Kingdom; 3 School of Sport, Health and Exercise Sciences, Bangor University, Bangor, United Kingdom; 4 Biarritz Olympique Rugby, Parc Des Sports Aguilera, Biarritz, France; Universidad Europea de Madrid, SPAIN

## Abstract

Reduced physical performance has been observed following the half-time period in team sports players, likely due to a decrease in muscle temperature during this period. We examined the effects of a passive heat maintenance strategy employed between successive exercise bouts on core temperature (T_core_) and subsequent exercise performance. Eighteen professional Rugby Union players completed this randomised and counter-balanced study. After a standardised warm-up (WU) and 15 min of rest, players completed a repeated sprint test (RSSA 1) and countermovement jumps (CMJ). Thereafter, in normal training attire (Control) or a survival jacket (Passive), players rested for a further 15 min (simulating a typical half-time) before performing a second RSSA (RSSA 2) and CMJ’s. Measurements of T_core_ were taken at baseline, post-WU, pre-RSSA 1, post-RSSA 1 and pre-RSSA 2. Peak power output (PPO) and repeated sprint ability was assessed before and after the simulated half-time. Similar T_core_ responses were observed between conditions at baseline (Control: 37.06±0.05°C; Passive: 37.03±0.05°C) and for all other T_core_ measurements taken before half-time. After the simulated half-time, the decline in T_core_ was lower (-0.74±0.08% vs. -1.54±0.06%, p<0.001) and PPO was higher (5610±105 W vs. 5440±105 W, p<0.001) in the Passive versus Control condition. The decline in PPO over half-time was related to the decline in T_core_ (r = 0.632, p = 0.005). In RSSA 2, best, mean and total sprint times were 1.39±0.17% (p<0.001), 0.55±0.06% (p<0.001) and 0.55±0.06% (p<0.001) faster for Passive versus Control. Passive heat maintenance reduced declines in T_core_ that were observed during a simulated half-time period and improved subsequent PPO and repeated sprint ability in professional Rugby Union players.

## Introduction

Rugby union is a high-intensity and intermittent collision sport which requires players to accelerate repeatedly between rucks to compete for possession [1]. Typically, two consecutive 40-min halves of rugby match-play are separated by a 10–15 minute half-time period. Although half-time is often considered crucial for primarily tactical reasons, physiologically, this pause in play can be viewed as a recovery period after the first half, a period of preparation before the second half and/or a transition between the two halves of play [2]. When compared to the opening phase of a game, intermittent sports players have demonstrated reduced exercise intensities during the initial stages of the second half [3,4]. Therefore, half-time interventions that seek to optimise performance in subsequent bouts of exercise, especially in the initial stages, are desirable.

Empirical observations highlight that the half-time practices currently employed by rugby players primarily include tactical discussion, provision of medical treatment and consumption of nutritional ergogenic aids; with half-time practices in soccer being similar [5]. However, periods of inactivity comparable in length to those observed during the rugby half-time period (i.e., ~15 min), elicit substantial physiological changes relating to acid-base balance [6], the glycaemic response [7,8,9] and muscle (T_m_) and core temperature (T_core_) changes [10,11]. Throughout the first half of a soccer match, Mohr et al. [10] observed increases in both T_m_ and T_core_. However, during a passive half-time period these T_m_ and T_core_ changes were not maintained as decreases in excess of 1°C occurred. The importance of changes in T_m_ on subsequent performance was established by Sargeant [12], who demonstrated that every 1°C reduction in T_m_ caused a 3% reduction in leg muscle power output. Findings from studies reporting attenuated losses of T_m_ and concomitant protection of physical performance [10,13] following an active re-warm-up further substantiate the importance of minimising body temperature losses during half-time.

However, intermittent sports players do not frequently use active re-warm up strategies in the applied setting [5]. Time constraints, a lack of co-operation from the coach/manager and a perceived negative impact upon the psychological preparations of players have been reported as barriers to the use of active rewarm-ups during half-time periods; this is despite practitioners acknowledging that attenuating losses in body temperature impact positively on subsequent exercise performance [5]. The high-collision nature of rugby means that considerable time will also be required for provision of medical attention during half-time. Therefore, half-time practices that are easily administered and which attenuate temperature loss and thus protect the temperature-related mechanisms that aid subsequent performance warrant further investigation.

Passive heat maintenance is a method used to attenuate reductions in body temperature [14]. Passive heat maintenance involves the use of specific methods (e.g., heated clothing, outdoor survival jackets, and heating pads) which seek to attenuate heat loss. Such strategies are easily applied to the desired muscle groups to maintain muscle temperature, and thus the temperature mediated pathways which aid performance [11]. For example, in professional Rugby Union players who wore a survival garment that incorporated a reflective surface designed to limit heat loss by radiation and convection in the time following the end of a warm-up (WU), repeated sprint performance and lower body peak power output (PPO) was greater than observed in a control trial [11]. Additionally, the decline in lower body PPO observed during the post-WU recovery period was related (r = 0.71) to the decline in T_core_ [11]. However, the efficacy of a passive heat maintenance strategy employed during a recovery period that separates consecutive bouts of high intensity exercise, such as half-time, remains to be established. Therefore, the aim of this study was to examine the influence of a heat maintenance strategy employed during a simulated half-time period on markers of T_core_, PPO and repeated sprint ability in professional Rugby Union players.

## Methods

### Participants

Following ethical approval from a university research ethics committee, 18 male professional Rugby Union players (age: 23 ± 1 years; height: 1.83 ± 0.05 m; body mass: 96.4 ± 8.7 kg) competing on behalf of a French top tier professional club volunteered to participate in this study. All players were informed of the potential risks associated with the study prior to giving their informed consent and in line with the recommendations of the team’s nutritionist were following a detailed diet plan which remained consistent between trials.

### Study design

The study accounted for circadian variability (i.e., trials were performed at the same time of the day ~10:00 h) and followed a randomised and counter-balanced repeated measures design. Each player completed a control and intervention trial which were separated by 7 days. Trials were carried out in a temperature controlled exercise physiology laboratory and an adjacent indoor sprint track (temperature: 22.0 ± 0.4°C; humidity: 50 ± 4%). Players reported for the trials at 10:00 h after consuming their typical training day breakfasts (replicated across trials) and having refrained from caffeine, alcohol and strenuous exercise in the 24 h preceding each trial. Upon arrival at the laboratory, players remained seated for 15 min while baseline T_core_ was measured and familiarisation instructions were discussed. After the WU players then remained at rest for 15 min (a time period representative of the duration separating the end of a WU and the start of competition in professional team sports) while wearing normal training attire before completing a repeated shuttle sprint ability (RSSA) test [15]. Notably, repeated sprint ability has been associated with activity rates during actual match-play [16] and can therefore be considered a key physical attribute to performance in Rugby Union.

As the influence of the heat maintenance strategy implemented during the simulated half-time break was the focus of the investigation, lower body explosive ability (i.e., CMJ performance) was assessed before and after the intervention (i.e., post-RSSA 1 and pre-RSSA 2). Therefore, upon completion of the first RSSA (RSSA 1), players carried out 3 countermovement jumps (CMJ). To replicate the half-time break in rugby match-play, players remained rested for a 15 min period while wearing normal training attire (Control) or a custom made survival jacket (Passive). A further three CMJ’s were performed before subsequently repeating a second RSSA test (RSSA 2).

All players were highly familiar with the RSSA and CMJ tests as these were part of the team’s testing battery and thus carried out on multiple occasions throughout the competitive year. The standardised WU was performed for ~25 min and was led by the team’s conditioning staff. The WU consisted of five repeats of ~40 m jogging, skipping and lateral bounding, before progressing to four repeats of ~30 m dynamic stretches focusing on the gluteals, quadriceps and hamstring muscle groups. Players then progressed on to plyometric strides (40 m x 2), high-knee striding into maximal sprinting (40 m x 2) and rolling start sprinting which progressively increased in intensity such that the final two repetitions were maximal (30 m x 5).

### Measurements

T_core_ was recorded at baseline, post-WU, pre-RSSA 1, post-RSSA 1 and pre-RSSA 2 using an ingestible temperature sensor (CorTemp Ingestible Core Body Temperature Sensor, HQ Inc, USA). The sensor transmitted a radio signal to an external receiver device (CorTemp Data Recorder, HQ Inc, USA), which subsequently converted the signal into digital format. Players ingested the sensor 3 h prior to the experimental trials and this method of T_core_ measurement has been demonstrated to be both reliable and valid [17].

PPO was determined using CMJ’s which were analysed on a portable force platform (Type 92866AA, Kistler, Germany) using methods described previously [18,19]. The vertical component of the ground reaction force (GRF) elicited during the CMJ and the participants’ body mass was used to determine the instantaneous velocity and displacement of the participant’s centre of gravity [20]. Instantaneous power output was the determined as per previous work from our group [19].

The RSSA test consisted of six 40 m (20 + 20 m separated by a 180° turn) shuttle sprints which were each separated by 20 s of passive recovery [15]. From a stationary start, the players commenced the test 0.3 m behind a line where electronic timing gates were placed (Brower TC-System, Brower Timing Systems, USA). Following instruction from the test administrator, the players sprinted 20 m and touched a second line with their foot before returning to the start line as quickly as possible. RSSA best, RSSA mean, and RSSA total were calculated according to Rampinini et al. [15].

### Intervention

The survival jacket (Blizzard Survival Jacket, Blizzard Protection Systems Ltd, UK) is made from materials designed to clinch the body, reduce convection, and trap warm, still air to provide insulation. The jacket also has a reflective surface which limits radiated heat loss [21]. The survival jackets used in the current study are similar to those used previously [11,14] and were custom made for athletes; tailored with long sleeves and were of a below the knee length.

### Statistical analysis

Statistical analyses were performed using SPSS software (Version 21; SPSS Inc., Chicago, IL) and data are presented as mean ± SEM. Significance was set at p≤0.05. Two-way repeated measures analysis of variance (ANOVA; within-subject factors: trial x time) were used where data contained multiple time points. Mauchly’s test was consulted and Greenhouse–Geisser correction was applied if sphericity was violated. Where significant p-values were identified for interaction effects (trial x time), trial was deemed to have influenced the response (given the similarity of the time points examined between trials) and simple main effect analyses were performed. Significant main effects of time were further investigated using pairwise comparisons with Bonferroni confidence-interval adjustment. Post-intervention relationships between changes in PPO and T_core_ were examined using Pearson’s product moment correlation coefficients.

## Results

**Fig 1:**
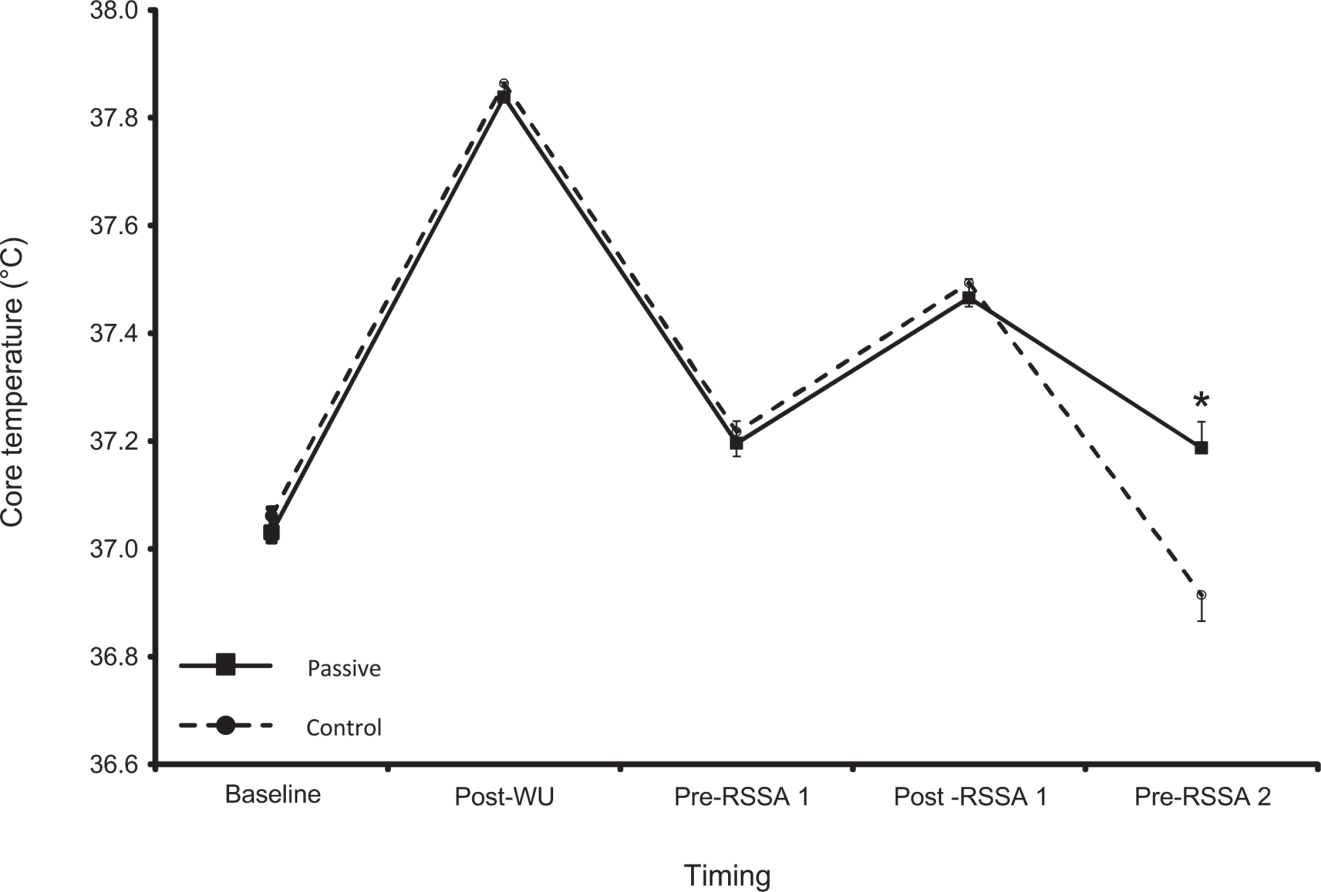
Mean ± SEM core temperature (T_core_) responses for both Control and Passive trials. * represents significant difference at p<0.001 level from Control at the same time point.

Fig. 1 illustrates the T_core_ responses and raw data is presented in S1 Table. A time x trial interaction (F_(1,25)_ = 72.528, p<0.001, partial-eta^2^ = 0.810) and main effect of time (F_(2,30)_ = 149.930, p<0.001, partial-eta^2^ = 0.898) was observed. Measurements of T_core_ were similar between conditions at baseline being 37.06±0.05°C and 37.03±0.05°C for Control and Passive respectively. At post-WU, increases in T_core_ were observed in both trials (Control, Passive: +2.17±0.09%, +2.18±0.09%, p<0.001). However, at pre-RSSA 1 elevated T_core_ was not maintained (p<0.001) but exercise subsequently increased T_core_ in both trials (p<0.001). At pre-RSSA 2, the decline in T_core_ observed in Passive was lower than Control (-0.74±0.08% vs. -1.54±0.06%, p<0.001).

**Fig 2:**
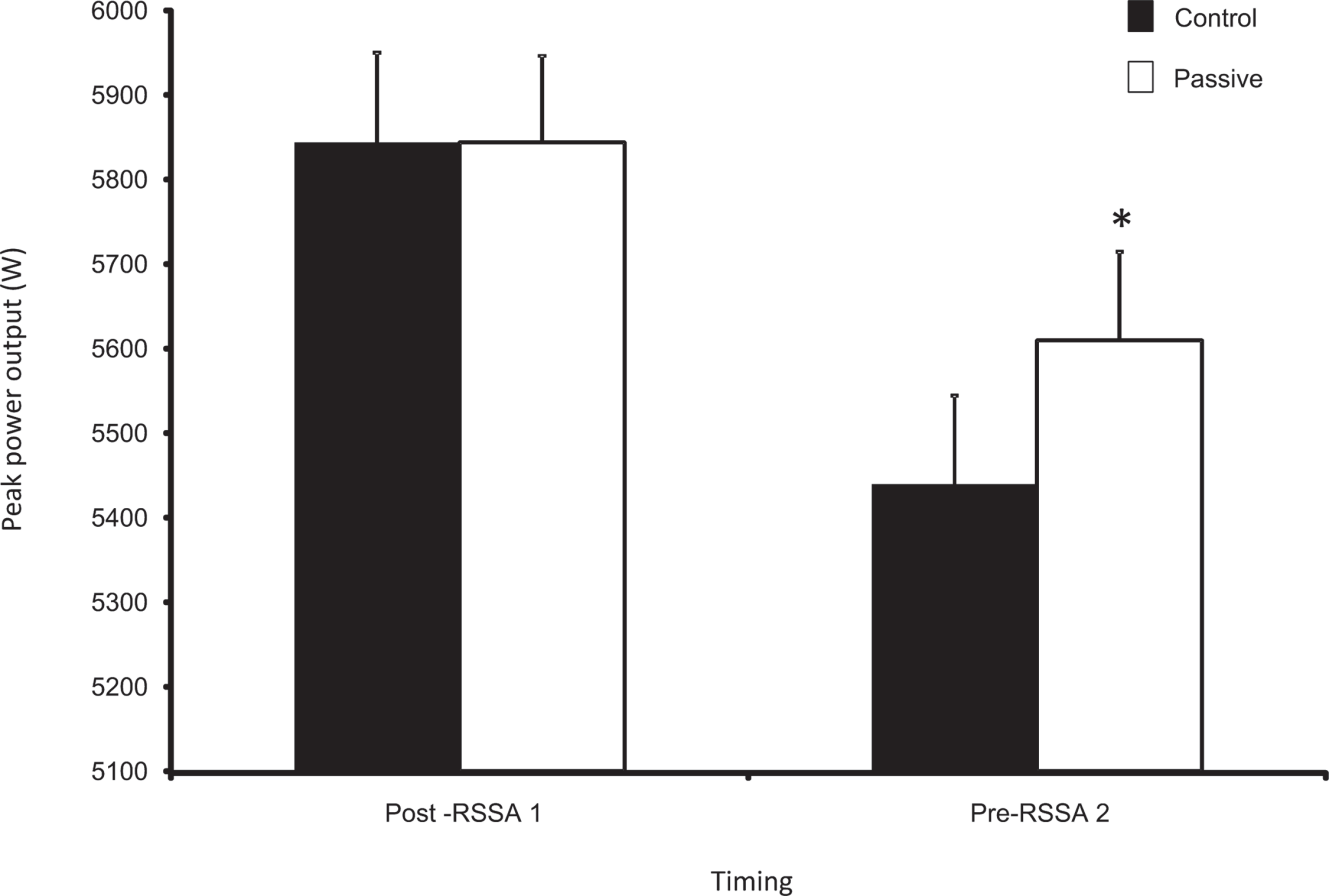
Mean ± SEM peak power output (PPO) for both Control and Passive trials. * represents significant difference at p<0.001 level from Control at the same time point.

Trial (time x trial interaction: F_(1,17)_ = 22.753, p<0.001, partial-eta^2^ = 0.572) and time (time effect: F_(1,17)_ = 290.183, p<0.001, partial-eta^2^ = 0.945) influenced PPO (Fig. 2; raw data included in S2 Table). Despite similar PPO values being observed between conditions at post-RSSA 1 (Control: 5844±106 W; Passive: 5844±102 W, p = 0.984); PPO was 3.18±0.65% higher in Passive versus Control at pre-RSSA 2 (Control: 5440±105 W; Passive: 5610±105 W, p<0.001). At pre-RSSA 2, the decline in PPO was significantly related to the decline in T_core_ (r = 0.632, p = 0.005).

**Fig 3:**
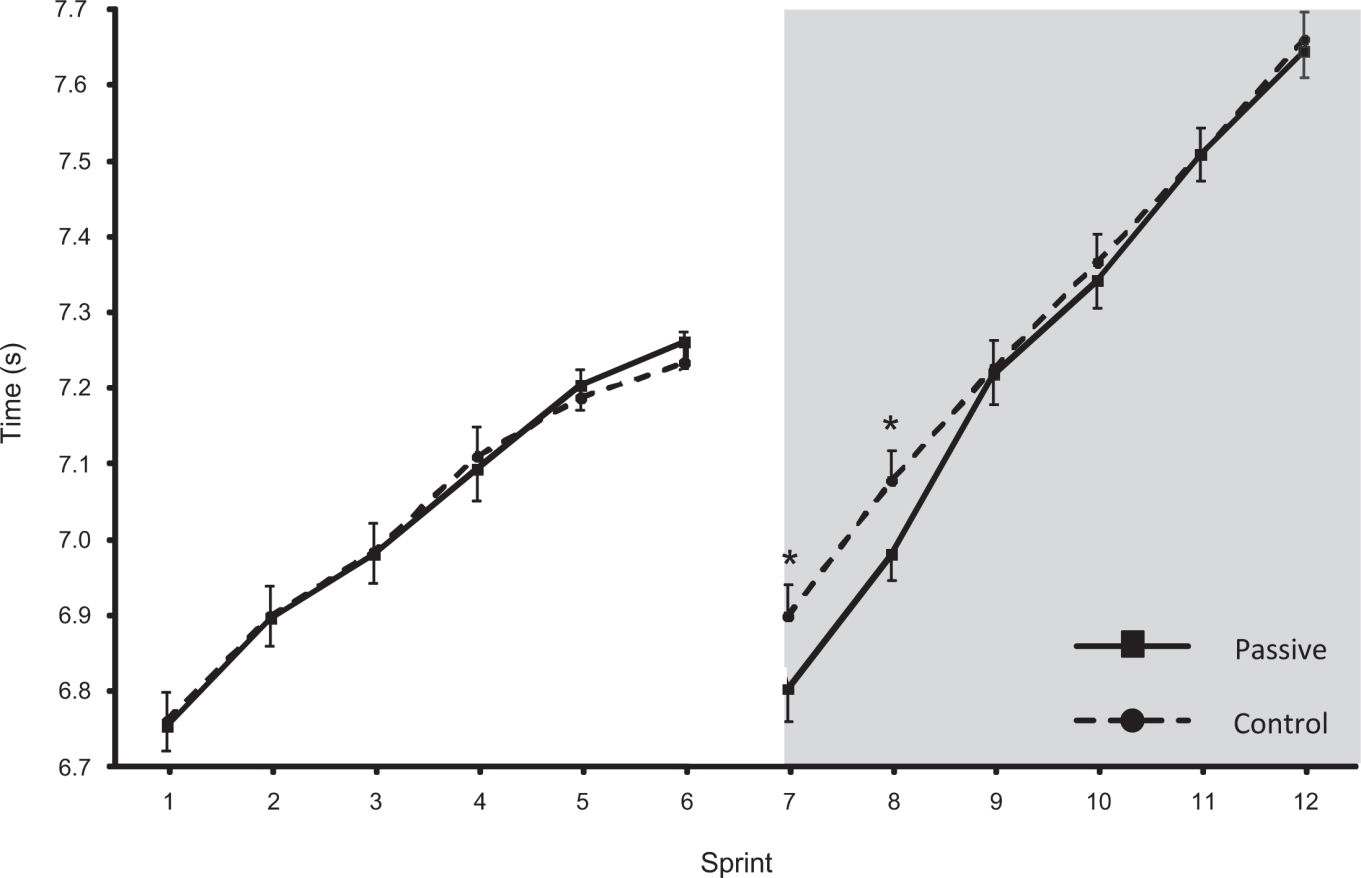
Mean ± SEM sprint times (s) during RSSA 1 (un-shaded region) and RSSA 2 (shaded region) for both Control and Passive conditions. * represents significant difference at p<0.001 level from Passive at the same time point.

Trial (time x trial interaction: F_(11,187)_ = 10.742, p<0.001, partial-eta^2^ = 0.387) and time (time effect: F_(3,49)_ = 276.559, p<0.001, partial-eta^2^ = 0.942) influenced performance in the 12 sprints performed (raw data presented in S3 Table). Although there were no differences between trials in RSSA 1, performance in the first two sprints of RSSA 2 were faster in Passive (Fig. 3). Consequently, RSSA best, RSSA mean and RSSA total were 1.39±0.17% (p<0.001), 0.55±0.06% (p<0.001) and 0.55±0.06% (p<0.001) faster in Passive versus Control during RSSA 2 (Table 1).

**Table 1:** Performance variables (Mean ± SEM) for each repeated sprint (RSSA) test performed pre and post half-time.

Variable	RSSA 1		RSSA 2	
	Control	Passive	Control	Passive
Best (s)	6.76 ± 0.04	6.75 ± 0.03	6.90 ± 0.04	6.80 ± 0.05 ^a^
Mean (s)	7.03 ± 0.03	7.03 ± 0.03	7.29 ± 0.03	7.25 ± 0.03 ^a^
Total (s)	42.18 ± 0.21	42.19 ± 0.20	43.74 ± 0.21	43.50 ± 0.20 ^a^

^a^ represents significant difference at P<0.001 level from Control at the same time point

## Discussion

This study examined the effects of a passive heat maintenance strategy employed during a simulated half-time period which separated consecutive bouts of repeated sprint exercise. Professional Rugby Union players who wore a survival jacket (Passive) throughout a simulated half-time period experienced lower reductions in core temperature (T_core_) over the 15 min and improved peak power output (PPO) and repeated sprint ability in subsequent exercise. We purport that in the Passive condition a greater preservation of temperature-related mechanisms explain the performance differences observed.

When no interventions are performed, T_core_ will decrease rapidly after the cessation of exercise [10,11,22]; a finding which was confirmed in our current study in both trials at pre-RSSA 1 (Fig. 1). Similarly, at pre-RSSA 2, T_core_ declined under both the Control and Passive conditions; however, the decline in T_core_ was less in the Passive versus Control trial as survival jackets were worn during the simulated half-time break. The garments used in this study have a reflective surface which acts to block radiated heat and its elastic properties cause the material to clinch the body which reduces convection [21]. In agreement with previous studies where such garments have been applied following a WU [11,14], our data demonstrates that this passive heat maintenance strategy lowered the reduction in T_core_ that occurred during the simulated half-time period. Therefore, applied practitioners should consider the use of survival jackets during the half-time period when seeking to minimise body temperature losses during periods of reduced activity.

During half-time, PPO reduced under both conditions (Fig. 2); however, at pre-RSSA 2, PPO was 3.18±0.65% higher in Passive versus Control (p<0.001). Moreover, the decline in PPO over half-time was significantly related to the decline in T_core_ during this period (r = 0.632, p = 0.005). Notably, every 1°C loss in T_m_ results in a 3% decrease in leg muscle power [12] and wearing insulated athletic trousers which incorporated a battery powered heating element reduced the drop in T_m_ throughout a 30 min period following a WU and improved subsequent sprint cycling PPO [23]. Although T_m_ was not measured in the current study, it is reasonable to suggest that the passive heat maintenance strategy employed would have also lessened the decline in T_m_ during this period as a similar time-course of T_core_ and T_m_ changes have been observed following the cessation of exercise [10].

Similarly, players performed better in RSSA 2 in Passive versus Control; particularly within the first two sprints, resulting in improvements in mean and total sprint times (Fig. 3 and Table 1). These responses may confer performance advantages to actions requiring sprinting and acceleration in the very initial stages of the second half. Under conditions of elevated body temperature, enhanced neural transmission rates in both peripheral and central nerves, and increased speeds of muscular contraction with concomitant decreases in both the time to peak tension and half relaxation time occur [24,25,26]. Additionally, elevated whole body temperatures increase phosphocreatine hydrolysis and glycolytic rates [27], through an up regulation of key glycolytic enzymes (e.g. phosphofructokinase and lactate dehydrogenase; [28]), which improves the capacity for ATP resynthesis and cross-bridge cycling [27,29]. It is therefore plausible that the improved performance observed during the initial stages of RSSA 2 reflected these temperature-related changes. In support of this supposition, T_core_ was higher at the pre-RSSA 2 time point in Passive versus Control and in agreement with previous authors [14], the reduced loss of T_core_ was significantly correlated with subsequent exercise performance.

Active re-warm ups incorporating moderate intensity running which commenced after seven minutes of a half-time recovery period attenuated a 1.5°C reduction in T_m_ and a 2.4% decrement in mean sprint performance observed in a control condition [10]. Intermittent agility exercise, whole body vibration, small sided games and lower body resistance exercises have also been used as active re-warm up strategies [13]. However, such strategies are not commonly employed in the field due to a number of barriers [5]. Here we present a passive heat maintenance strategy which is likely to have minimal impact upon existing half-time practices while reducing body temperature losses and in doing so eliciting ergogenic performance effects in subsequent exercise.

The lack of measurement of T_m_ in this study may be viewed as a limitation; however, T_m_ measurement was not feasible due to the nature of the study design and the recruitment of professional athletes to the study. The measurement of T_core_ as opposed to T_m_ does not detract from the meaningfulness of the data reported, as both temperature markers have been shown to influence performance [22], and to show similar time-course patterns of response [10]. Also, the use of the RSSA test in this study may be questioned; however, the use of such a protocol standardises the physiological demands elicited between repeated trials making the effects of exercise repeatable (demonstrated by the pre-intervention responses to RSSA 1). Moreover, repeated sprint ability is associated with activity rates during actual match-play in Rugby Union players [16]. Finally, it could be speculated that the use of the survival jacket during the half-time period may have led to a placebo effect. That said, the differences in T_core_ observed at the pre-RSSA 2 time point (Fig. 1) and the growing body of literature supporting the ergogenic effects of passive heat maintenance [11,14,22], support the notion that temperature-related physiological mechanisms, as opposed to a placebo effect, explain these responses. While acknowledging the impact of these limitations, applied practitioners should consider the use of passive heat maintenance strategies to reduce losses of body temperature that occur during the half-time period and thus improve subsequent exercise performance.

In summary, numerous authors have demonstrated that an active rewarm-up of intermittent sports players can attenuate losses of T_core_ and T_m_ during the half-time period [10,13]. However, in applied settings the feasibility of such practices may be limited despite the acknowledged benefits of attenuating body temperature losses for subsequent exercise performance [30]. We have demonstrated that a 15-min period results in a loss of body temperature which impairs subsequent physical performances that require PPO and repeated sprint ability. However, use of a passive heat maintenance strategy, albeit in a controlled, laboratory orientated protocol, effectively reduces the drop in T_core_ that occurs during a simulated half-time period, and thus helps ameliorate temperature-related declines in performance. Future research opportunities exist to examine the use of passive heat maintenance strategies employed during the half-time period on performances elicited during actual competitive match-play.

## Conclusion

In conclusion, the effects of a passive heat maintenance strategy administered during a simulated half-time period on physiological (i.e., T_core_) and performance (i.e., PPO and repeated sprint ability) responses was assessed in a group of professional Rugby Union players. Our data demonstrates that the application of a survival jacket during a simulated half-time period improved subsequent physical performances that required PPO and repeated sprint ability. Such findings are likely due to minimised losses in body temperature occurring during the recovery period that separated the two consecutive bouts of exercise.

## Supporting Information

S1 Table Raw core temperature (T_core_) data. (XLSX)

S2 Table Raw peak power output (PPO) data. (XLSX)

S3 Table Raw sprint times. (XLSX)

## References

[1] Deutsch MU, Kearney GA, Rehrer NJ (2007) Time—motion analysis of professional rugby union players during match-play. J Sports Sci 25: 461–472. 17365533 10.1080/02640410600631298

[2] Sugiura K, Kobayashi K (1998) Effect of carbohydrate ingestion on sprint performance following continuous and intermittent exercise. Med Sci Sports Ex 30: 1624–1630. 9813876 10.1097/00005768-199811000-00011

[3] Weston M, Batterham AM, Castagna C, Portas MD, Barnes C, Harley J, et al (2011) Reduction in physical match performance at the start of the second half in elite soccer. Int J Sports Physiol Perform 6: 174–182. 21725103 10.1123/ijspp.6.2.174

[4] Bradley PS, Sheldon W, Wooster B, Olsen P, Boanas P, et al (2009) High-intensity running in English FA Premier League soccer matches. J Sports Sci 27: 159–168. 10.1080/02640410802512775 19153866

[5] Towlson C, Midgley AW, Lovell R (2013) Warm-up strategies of professional soccer players: practitioners' perspectives. J Sports Sci 31: 1393–1401. 10.1080/02640414.2013.792946 23734830

[6] Russell M, Kingsley M (2012) Changes in Acid-base balance during simulated soccer match play. J Strength Cond Res 26: 2593–2599. 10.1519/JSC.0b013e31823f284e 22067253

[7] Russell M, Benton D, Kingsley M (2012) Influence of carbohydrate on skill performance during a soccer simulation. J Sci Med Sport 15: 348–354. 10.1016/j.jsams.2011.12.006 22230353

[8] Russell M, Benton D, Kingsley M (2014) Carbohydrate ingestion before and during soccer match-play and blood glucose and lactate concentrations J Ath Train 49: 447–453. 10.4085/1062-6050-49.3.12 PMC4151832 24933430

[9] Russell M, Rees G, Benton D, Kingsley M (2011) An exercise protocol that replicates soccer match-play. Int J Sports Med 32: 511–518. 10.1055/s-0031-1273742 21472627

[10] Mohr M, Krustrup P, Nybo L, Nielsen JJ, Bangsbo J (2004) Muscle temperature and sprint performance during soccer matches—beneficial effect of re-warm-up at half-time. Scand J Med Sci Sports 14: 156–162. 15144355 10.1111/j.1600-0838.2004.00349.x

[11] Kilduff LP, West DJ, Williams N, Cook CJ (2014) The influence of passive heat maintenance on lower body power output and repeated sprint performance in professional rugby league players. J Sci Med Sport 16: 482–486. 10.1016/j.jsams.2012.11.889 23246444

[12] Sargeant AJ (1987) Effect of muscle temperature on leg extension force and short-term power output in humans. Eur J Appl Physiol Occup Physiol 56: 693–698. 3678224 10.1007/BF00424812

[13] Lovell R, Midgley A, Barrett S, Carter D, Small K (2013) Effects of different half-time strategies on second half soccer-specific speed, power and dynamic strength. Scand J Med Sci Sports 23: 105–113. 10.1111/j.1600-0838.2011.01353.x 21812822

[14] Cook C, Holdcroft D, Drawer S, Kilduff LP (2013) Designing a warm-up protocol for elite bob-skeleton athletes. Int J Sports Physiol Perform 8: 213–215. 22904126 10.1123/ijspp.8.2.213

[15] Rampinini E, Bishop D, Marcora SM, Ferrari Bravo D, Sassi R, Impellizzeri FM (2007) Validity of simple field tests as indicators of match-related physical performance in top-level professional soccer players. Int J Sports Med 28: 228–235. 17024621 10.1055/s-2006-924340

[16] Smart D, Hopkins WG, Quarrie KL, Gill N (2014) The relationship between physical fitness and game behaviours in rugby union players. Eur J Sports Sci 14: S8–S17. 10.1080/17461391.2011.635812 24444248

[17] Byrne C, Lim CL (2007) The ingestible telemetric body core temperature sensor: a review of validity and exercise applications. Br J Sports Med 41: 126–133. 17178778 10.1136/bjsm.2006.026344 PMC2465229

[18] West DJ, Owen NJ, Jones MR, Bracken RM, Cook CJ, Cunningham DJ et al (2011) Relationships between force-time characteristics of the isometric midthigh pull and dynamic performance in professional rugby league players. J Strength Cond Res 25: 3070–3075. 10.1519/JSC.0b013e318212dcd5 21993026

[19] Owen NJ, Watkins J, Kilduff LP, Bevan HR, Bennett M (2014) Development of a criterion method to determine peak mechanical power output in a countermovement jump. J Strength Cond Res 28: 1552–1558. 10.1519/JSC.0000000000000311 24276298

[20] Hatze H (1998) Validity and reliability of methods for testing vertical jumping performance. J Appl Biomech 14: 127–140.

[21] Allen PB, Salyer SW, Dubick MA, Holcomb JB, Blackbourne LH (2010) Preventing hypothermia: comparison of current devices used by the US Army in an in vitro warmed fluid model. J Trauma 69: S154–S1561. 10.1097/TA.0b013e3181e45ba5 20622611

[22] West DJ, Dietzig BM, Bracken RM, Cunningham DJ, Crewther BT, Cook CJ et al (2013) Influence of post-warm-up recovery time on swim performance in international swimmers. J Sci Med Sport 16: 172–176. 10.1016/j.jsams.2012.06.002 22789310

[23] Faulkner SH, Ferguson RA, Gerrett N, Hupperets M, Hodder SG, Havenith G (2013) Reducing muscle temperature drop after warm-up improves sprint cycling performance. Med Sci Sports Ex 45: 359–365. 10.1249/MSS.0b013e31826fba7f 22935735

[24] Hill DK (1972) Resting tension and the form of the twitch of rat skeletal muscle at low temperature. J Physiol 221: 161–171. 5016979 10.1113/jphysiol.1972.sp009746 PMC1331327

[25] Bennett AF (1984) Thermal dependence of muscle function. Am J Physiol 247: R217–R229. 6380314 10.1152/ajpregu.1984.247.2.R217

[26] Davies CT, Young K (1983) Effect of temperature on the contractile properties and muscle power of triceps surae in humans. J Appl Physiol Respir Environ Exerc Physiol 55: 191–195. 6885568 10.1152/jappl.1983.55.1.191

[27] Edwards RH, Harris RC, Hultman E, Kaijser L, Koh D, Nordesjo LO (1972) Effect of temperature on muscle energy metabolism and endurance during successive isometric contractions, sustained to fatigue, of the quadriceps muscle in man. J Physiol 220: 335–352. 5014103 10.1113/jphysiol.1972.sp009710 PMC1331706

[28] Macdonald M, Pedersen PK, Hughson RL (1997) Acceleration of VO2 kinetics in heavy submaximal exercise by hyperoxia and prior high-intensity exercise. J Appl Physiol 83: 1318–1325. 9338442 10.1152/jappl.1997.83.4.1318

[29] Febbraio MA, Carey MF, Snow RJ, Stathis CG, Hargreaves M (1996) Influence of elevated muscle temperature on metabolism during intense, dynamic exercise. Am J Physiol 271: R1251–R1255. 8945960 10.1152/ajpregu.1996.271.5.R1251

[30] Russell M, West DJ, Harper LD, Cook CJ, Kilduff LP (2014) Half-time strategies to enhance second-half performance in team-sports players: A review and recommendations. Sports Med doi: 10.1007/s40279-014-0297-0 25504550

